# Detoxification in a structured programme is effective for so-called refractory medication-overuse headache

**DOI:** 10.1186/1129-2377-14-S1-P150

**Published:** 2013-02-21

**Authors:** SB Munksgaard, L Bendtsen, RH Jensen

**Affiliations:** 1 Danish Headache Center, Dept. of Neurology, Glostrup Hospital, Faculty of Health Sciences, University of Copenhagen, Denmark

## Introduction

The strategy regarding whether detoxification for medication overuse headache (MOH) is needed or not has been heavily debated. Patients are often regarded as treatment resistant if they fail one withdrawal attempt. Further, many report a substantial relapse to MOH within the first year after withdrawal.

## Objective

To evaluate the long-term efficacy of two different treatment programmes for MOH in so-called treatment-resistant patients.

## Methods

MOH patients who had previously been unsuccessfully treated by neurologists were enrolled in one of 2 structured detoxification programmes in a tertiary headache centre: A) a one-week withdrawal with restricted analgesics, rescue medications and prophylactics from Day 1 followed by advice of restricted intake of symptomatic medications or B) a 2-month drug-free period and multidisciplinary education in groups and subsequent initiation of restricted symptomatic medication and prophylactics as required. All patients were closely followed up for a year.

## Results

86 of 98 patients completed the 12-month follow-up. Totally, headache frequency was reduced by 39% (p<0.001), medication use by 63% (p<0.001) and 83% remained cured of MOH. Headache frequency was reduced with more than 50% in 42 patients (49%) and 52 (61%) reverted to episodic headache, and with no difference between the groups. Patients in programme B used significantly less symptomatic medication: 6.5 days/4 weeks compared with 8.7 days/4 weeks in programme A (p=0.02), and the 56% of patients in programme B who needed prophylactic medication was significantly less than the 80% in programme A (p=0.02). Further, programme B required fewer resources from the staff.

## Conclusion

Structured detoxification with close follow-up by a multidisciplinary team for one year is highly effective in patients with previously treatment-resistant MOH. We recommend a multidisciplinary educational programme for patients in groups due to cost-effectiveness and limited use of medication.

